# Effect of RaCe, ProTaper, and V-Taper rotary systems on dentinal crack formation during endodontic treatment: An *in vitro* study

**DOI:** 10.34172/joddd.2021.041

**Published:** 2021-12-05

**Authors:** Shahriar Shahi, Mahmoud Bahari, Mohammad Samiei, Hamidreza Yavari, Shabnam Mohammadzadeh

**Affiliations:** ^1^Department of Endodontics, Faculty of Dentistry, Tabriz University of Medical Sciences, Tabriz, Iran; ^2^Dental and Periodontal Research Centre and Department of Operative Dentistry, Faculty of Dentistry, Tabriz University of Medical Sciences, Tabriz, Iran; ^3^Dentist, General Practitioner, Tabriz, Iran

**Keywords:** Crack, Dentin, Instrumentation, Root canal

## Abstract

**Background.** Prevention of dentinal crack formation is of utmost importance in endodontic treatment. This study aimed to evaluate the effect of RaCe, ProTaper, and V-Taper rotary systems on dentinal crack formation in three root regions during endodontic treatment.

**Methods.** Seventy human mandibular first molars were selected randomly, and their distal roots were used. Ten samples were assigned to the control group, and sixty samples were assigned to three groups (n=20). Each group was prepared with RaCe, ProTaper, or V-Taper rotary files according to the manufacturers’ instructions. A stereomicroscope was used to view cracks at ×40 magnification. Friedman’s, chi-squared, and Kruskal-Wallis tests were used for statistical analysis of data (*P* < 0.05).

**Results.** A comparison of the three rotary systems did not reveal any significant differences in the number of cracks between the three root thirds evaluated (*P* > 0.05). A comparison of the number of cracks in the coronal, middle, and apical thirds in each rotary system showed that the number of cracks in the middle third was fewer than that in the two other thirds only in the V-Taper group (*P* < 0.05), with no significant differences in the ProTaper and RaCe groups between the different root regions (*P* > 0.05).

**Conclusion.** The application of RaCe, ProTaper, and V-Taper rotary files resulted in a similar rate of crack formation in endodontic treatment. V-Taper files created the minimum number of cracks in the middle third.

## Introduction

One of the essential steps in root canal treatment is root canal preparation for proper debridement of the root canal and provision of a favorable space for compacting the obturation material.^1^ The aim is to eliminate all the residual pulpal tissue, bacteria, and biological products and create a space within the root canal to make it possible to obturate the root canal properly.^2,3^ Root canals can be prepared with manual or rotary instruments. Manual techniques are time-consuming, and in curved root canals, they might lead to procedural errors, such as ledge formation or root canal transportation due to their poor flexibility. Ni-Ti files can decrease procedural errors during root canal preparation in curved root canals due to their excellent flexibility.^4^ The superelastic properties of Ni-Ti files allow them to return to their initial shape after their deformation, unlike stainless steel files. Besides, the abrasion and deformation of these files are less than stainless steel files. In addition, Ni-Ti files have superb anti-corrosive properties. These properties make these files more flexible, increasing their adaptation with the root canal curvature and fracture resistance.^5^

Furthermore, clinically, it is very difficult and tiresome to use hand files in slender root canals.^6-8^ Root canal preparation might lead to dentinal cracks, finally leading to vertical root fracture (VRF).^9,10^ Since VRF leads to tooth extraction in almost all cases, it is of utmost importance to prevent crack formation during endodontic treatment. Various rotary systems have been introduced in recent years. These systems save time, are appealing to users, and are safe during root canal preparation.^11^

ProTaper rotary files are the most commonly used files for root canal treatment. These files remove more dentin from the coronal third than other file systems by a gradual increase in their taper and active cutting movements.^12^ It has been reported that the ProTaper rotary file causes more dentin damage than other rotary instruments.^13^ RaCe rotary files have a non-cutting tip and a triangular cross-section. Intermittent cutting edges prevent torsional effect; they also have very low torque as an advantage.^14^ V-Taper files have been introduced recently and are compatible with most of the rotary motors on the market. They have strong mechanical properties and adequate flexibility to prevent root canal transportation; however, they are strong enough for effective cutting. In addition, they are very resistant to cyclic fatigue.

Capar et al^11^ evaluated the effect of ProTaper Universal (PTU), ProTaper Next, and Hyflex files on dentinal cracks and reported no dentinal cracks in the control group, with no VRFs in any group. ProTaper Next and Hyflex exhibited fever cracks (28%) than the PTU files (56%). Ceyhanli et al^15^ carried out an ex vivo study on the emergence of dentinal cracks in the root after preparation of the root canal system with the PTU, RaCe, and Safesider systems and reported significantly higher microcracks in the PTU (42%), Safesider (35%), and RaCe (25%) systems than the control samples.

Despite the availability of many studies on the effect of different rotary files on dentinal cracks, no study has evaluated and compared the newly introduced V-Taper system with previous files. Therefore, the present study aimed to assess the effect of ProTaper, RaCe, and V-Taper files on the induction of dentinal cracks in tooth roots.

## Methods

The tooth samples used in the present study were randomly selected from extracted human mandibular first molars. The distal roots of the teeth were used. The inclusion criteria consisted of freshly extracted teeth with one root canal in the distal root, a root length of 10-12 mm, no visible fracture lines in the teeth, closed apices, no anatomic anomalies, no previous root canal treatment, no internal or external resorption, and no calcification.

Seventy teeth were included in the present study. The selected samples were transversely cut at CEJ. Then, the distal root was separated from the other roots using a vertical cut. After cutting and separating the distal root, the root surfaces of all the samples were evaluated under a loupe and light (HEINE Optotechnik GmbH & Co.KG Herrsching, Germany) at ×2.5 magnification. Samples with craze lines were excluded from the study. The samples were first disinfected in 2.5% NaOCl for 10 minutes and then assigned into four groups. The samples were stored in the physiologic serum in all the stages of the study.

Group 1 (n = 10) was considered the control group. The samples in this group remained intact throughout the study. The remaining 60 samples were assigned to the ProTaper, RaCe, and V-Taper groups (n = 20).

The working length (WL) in all three groups was determined with a #15 stainless steel hand file. After the file tip became visible at the root apex, the file length was measured, and 0.5 mm shorter than this length was deemed the WL. The samples in which the WL was not easily achieved with a #20 file were excluded to standardize the samples in terms of the root canal space.

The samples in group 1 were prepared with RaCe files (FKG Dentaire La-Chaux-de-Fonds, Switzerland). The rotating speed of the machine was adjusted to 600 rpm. Besides, the torque was set to 2 Ncm. The samples in group 2 were prepared with the ProTaper rotary system (Dentsply Maillefer, Ballaigues, Switzerland). The rotating speed of the machine was set at 300 rpm with a 2-Ncm torque. The samples in this group were prepared with SX, S1, S2, F1, F2, and F3 files, respectively. The first three files were used for shaping, and the next three files were used to finish the root canal treatment. The samples in group 3 were prepared with the V-Taper rotary system (Fanta Dental Materials Co., Shanghai, China). The rotation speed and torque were set at 350 rpm and 2 Ncm, respectively. The files used in this group and system consisted of AFBS1: #17/12, AFBS2: #18/05, AFBS3: #25/66, and AFBS4: #35/06. At each stage of file change, the root canal was irrigated with normal saline solution to remove the debris. Furthermore, before applying each file, the file tip was coated with RC-Prep to better glide of the file within the root canal. The prepared samples were first embedded in rectangular wax boxes filled with transparent acrylic resin, which resulted in adequate stability of the samples during the cutting procedures. The acrylic resins were fixed on the special plates and cut transversely with a cutting machine. The width of each sectioned sample was 1 mm. Copious water was used for cooling the cutting blade during the cutting procedure. The teeth in each group were separately evaluated in the coronal, middle, and apical thirds for the possible cracks on the samples. A stereomicroscope (TN-PSE30, Nikon, Tokyo, Japan) was used to visualize cracks at ×40 magnification.

### Statistical analysis

Friedman’s, chi-squared, and Kruskal-Wallis tests were used to compare the mean number of cracks between the apical, middle, and coronal thirds in each rotary system, and the cracks between the study groups (control, V-Taper, ProTaper, and RaCe), respectively. SPSS 24 was used for the analyses of data at a significance level of *P* < 0.05.

## Results

Table 1 presents the number of cracks in the apical, middle, and cervical thirds of the roots prepared with the three rotary systems.

**Table 1:** The number of cracks in the roots prepared with the three rotary systems in the apical, middle, and cervical thirds of the roots

**Group**	**Root region**	**Number of samples**	**Number of cracked teeth**	**Number of cracks**	**Mean**	***P*** ** value**
Control	Apical third	10	3	3	0.30	0.17
	Middle third	10	1	1	0.10	
	Coronal third	10	0	0	0.00	
V-TAPER	Apical third	20	7	9	0.45	0.045
	Middle third	20	2	2	0.10	
	Coronal third	20	4	5	0.25	
RaCe	Apical third	20	6	9	0.45	0.27
	Middle third	20	4	4	0.20	
	Coronal third	20	9	9	0.45	
Pro-Taper	Apical third	20	6	8	0.40	0.39
	Middle third	20	4	4	0.20	
	Coronal third	20	5	9	0.45	

### The number of cracks in the apical third

In 30%, 35%, and 30%, and 30% of the samples in the control, V-Taper, RaCe, and ProTaper groups, respectively, at least one crack was found in the apical area. According to chi-squared and Kruskal-Wallis tests, the rotary system type did not significantly affect crack formation in the apical area (*P* = 0.98).

### The number of cracks in the middle third

In 10%, 10%, 20%, and 20% of the samples in the control, V-Taper, and RaCe, and ProTaper group, respectively, there was at least one crack in the middle third. According to chi-squared and Kruskal-Wallis tests, the rotary system type did not significantly affect crack formation in the middle third (*P* = 0.73).

### The number of cracks formed in the coronal third

No cracks were detected in the coronal third of the samples in the control group; however, in 20%, 45%, and 25% of the samples in the V-Taper, RaCe, and ProTaper groups, respectively, at least one crack was found in the coronal third. According to chi-squared and Kruskal-Wallis tests, the rotary system type did not significantly affect crack formation in the middle third (*P* = 0.73).

### Comparison of the number of cracks between the three root regions in each rotary system

- There were three, one, and no cracks in the apical, middle, and coronal thirds, respectively, in the control group. Besides, the means of cracks in these regions were 0.30, 0.10, and none, respectively. Friedman’s test did not reveal any significant differences in the means of cracks formed between the three regions (*P* = 0.17).
- There were nine, two, and five cracks in the apical, middle, and coronal thirds, respectively, in the V-Taper rotary system group. In addition, the means of cracks in these regions were 0.45, 0.10, and 0.25, respectively. Friedman’s test revealed significant differences in the means of cracks formed between these regions (*P* = 0.04).
- There were nine, four, and nine cracks in the apical, middle, and coronal thirds, respectively, in the RaCe rotary system group. The means of cracks in these regions were 0.45, 0.20, and 0.45, respectively. Friedman’s test did not reveal any significant differences in the means of cracks formed between these regions (*P* = 0.27).
- There were eight, four, and nine cracks in the apical, middle, and coronal thirds, respectively, in the ProTaper rotary system. The means of cracks in these regions were 0.40, 0.20, and 0.45, respectively. Friedman’s test did not reveal any significant differences in the means of cracks formed between these regions (*P* = 0.39).

## Discussion

One of the new achievements widely used in endodontic treatment is the Ni-Ti alloy. The elastic properties of this alloy make it very useful in removing debris from the root canals with great curves. This property also preserves the root canal’s initial shape while effectively removing or minimizing vital and necrotic pulp tissue and microorganisms in the root canal system.^1^ Rotary files can follow the root canal path, do not exert excessive stress, and are a proper choice for instrumentation.^6,16^ This system is superior to the manual and conventional system because it can adapt to the canal shape when the file rotates within the root canal. However, the rotary systems have some disadvantages, too, including crack formation during root canal treatment. These cracks possibly form due to the file’s physical properties. Since rotary files create more cracks than hand files, different manufacturers have introduced various files to minimize the drawbacks of their products.^17^

The chief aim of the present study was to compare the behavior of three commercially available files in terms of crack formation or increasing the number of cracks in different regions of the root. The results showed no significant differences in crack formation in the apical, middle, and coronal thirds between the different study groups. Previous studies have evaluated the effects of different systems on the possible increase in the incidence of VRFs. Most of these studies have emphasized the impact of file design and taper on crack formation.^18-20^ Kim et al^21^ reported that differences in the design of different systems are responsible for differences in crack formation and the final VRF. They compared ProTaper (with a triangular cross-section), Profile (u-shaped cross-section), and Light-Speed (round cross-section) systems and concluded that instrument design could significantly affect stress concentration in the root apical area followed by the crack formation in this area. They showed that a triangular design in the file cross-section induced maximum tensile and shearing stress in the apex; however, the round cross-section exerted a minimum amount of stress.

In the present study, no significant differences were detected in crack formation, which might be attributed to the relative similarity between the three files’ cross-sections, consisting of triangular cross-section (RaCe), modified triangular cross-section (ProTaper), and parabolic cross-section (V-Taper). In contrast to the present study results, some previous studies have shown that the PTU systems result in more damage to the tooth structure. This might be because, in the PTU system, large finishing files are used to prepare the apical area, and due to the greater thickness of finishing files up to around 0.09 mm, more stress is exerted on the root canal walls, resulting in crack formation.^18^ Capar et al^11^ evaluated ProTaper Next, PTU, and Hyflex files regarding crack formation in the apical area. There were cracks in the control group, too, and the most numerous cracks were detected in the PTU group. The two other files were not significantly different from each other in crack formation.

Liu et al^17^ evaluated K3, PTU, and Flex K files and concluded that crack propagation was much faster in the apical area. However, there were no significant differences between the different areas (coronal, middle, and apical) in crack formation in the present study, with the fewest cracks in the middle third in the V-Taper group. Ceyhanli et al^15^ compared PTU, RaCe, and Safesider files. The unprepared samples, too, exhibited some cracks. However, in contrast to the present study, these files increased the cracks compared to the control group, with the most numerous cracks in the PTU file group.

Zhou et al^22^ evaluated the cracks formed by Twisted File, WaveOne, PTU, and Twisted File Adaptive systems in small and large root canals. One of the most important results was that all the files created more cracks in small root canals. In the present study, the samples were standardized regarding the root canal space, which might have affected the lack of significant differences in crack formation between the rotary systems used in the present study. Kesim et al^23^ evaluated K-files as a control group with K3XF rotary file with direct rotation and ProTaper Next and Twisted File Adaptative files with direct and reciprocal rotations. The results showed no significant differences in the number of cracks between the rotary systems and K-files at a distance of 3 mm from the apex. However, at 3- and 9-mm distances from the apex, ProTaper Next and Twisted File Adaptive files created more cracks than the other groups. In the present study, too, the preparation of the samples with the V-Taper system resulted in fewer cracks in the middle third.

In the present study, although there were no significant differences in crack formation between the three rotary file systems, overall, the V-Taper system exhibited a better performance in the middle third than the two other systems. Since the V-Taper files are a new file system on the market, adequate studies are not available, and further studies are suggested on these files.

## Conclusion

The most important conclusions of the present study are:

- All three file systems created cracks during root canal treatment.
- There were no significant differences in crack formation between the apical, middle, and coronal thirds.
- ProTaper and RaCe files resulted in a similar crack formation rate in the three regions of the root; however, V-Taper files created fewer cracks in the middle third.

## Authors’ Contributions

MB and ShSh planned this study. The literature review was performed by ShSh, MB, YR, MS, HRY, and ShM. MB and ShM performed the experiments. SM and HRY carried out the statistical analyses and interpretation of data. MB and HRY drafted the manuscript. All the authors critically revised the manuscript for intellectual content. All the authors have read and approved the final manuscript.

## Acknowledgments

The authors extend their appreciation to the Dental and Periodontal Research Centre at the Office of Vice Chancellor for Research and Technology, Tabriz University of Medical Sciences, for the financial support of this research.

## Funding

The study was supported by the Dental and Periodontal Research Center at the Faculty of Dentistry, Tabriz University of Medical Sciences.

## Competing Interests

The authors declare no competing interests with regards to the authorship and/or publication of this article.

## Ethics Approval

The study protocol was approved by the local ethics committee under the code IR.TBZMED.REC.1398.594.
